# Calpain: a molecule to induce AIF-mediated necroptosis in RGC-5 following elevated hydrostatic pressure

**DOI:** 10.1186/1471-2202-15-63

**Published:** 2014-05-12

**Authors:** Lei Shang, Ju-Fang Huang, Wei Ding, Shuang Chen, Li-Xiang Xue, Ruo-Fei Ma, Kun Xiong

**Affiliations:** 1 Department of Anatomy and Neurobiology, Central South University School of Basic Medical Sciences, 172 Tongzi Po Road, 410013 Changsha, Hunan, China; 2 Department of Biochemistry and Molecular Biology, Peking University Health Science Center, 100191 Beijing, China; 3 Xiangya School of Medicine, Central South University, 410013 Changsha, Hunan, China

**Keywords:** Retinal ganglion cells-5, Calpain, Elevated hydrostatic pressure, tAIF, Necroptosis

## Abstract

**Background:**

RIP3 (Receptor-interacting protein 3) pathway was mainly described as the molecular mechanism of necroptosis (programmed necrosis). But recently, non-RIP3 pathways were found to mediate necroptosis. We deliberate to investigate the effect of calpain, a molecule to induce necroptosis as reported (Cell Death Differ 19:245–256, 2012), in RGC-5 following elevated hydrostatic pressure.

**Results:**

First, we identified the existence of necroptosis of RGC-5 after insult by using necrostatin-1 (Nec-1, necroptosis inhibitor) detected by flow cytometry. Immunofluorescence staining and western blot were used to detect the expression of calpain. Western blot analysis was carried out to describe the truncated AIF (tAIF) expression with or without pretreatment of ALLN (calpain activity inhibitor). Following elevated hydrostatic pressure, necroptotic cells pretreated with or without ALLN was stained by Annexin V/PI, The activity of calpain was also examined to confirm the inhibition effect of ALLN. The results showed that after cell injury there was an upregulation of calpain expression. Upon adding ALLN, the calpain activity was inhibited, and tAIF production was reduced upon injury along with the decreased number of necroptosis cells.

**Conclusion:**

Our study found that calpain may induce necroptosis *via* tAIF-modulation in RGC-5 following elevated hydrostatic pressure.

## Background

Calpains are calcium-activated neutral protease, which belongs to the family of cytosolic cysteine proteinases. They form heterodimers which are composed of a large 80 kDa catalytic subunit and a common 30 kDa regulatory subunit [1]. The calpains are widely distributed in most of the mammal tissues. The calpains are also implicated in physiological and pathophysiological processes, such as cytoskeletal reorganization, signal transduction pathways, cell cycle regulation and certain apoptosis pathways. The dysfunction of calpains is related to certain diseases like cataract, Parkinson’s disease and Alzheimer’s disease [2-5].

During cerebral hypoxic-ischemia, there’s an overload of intracellular calcium which activates calpains, as a result neuronal apoptosis is triggered *via* caspase-3 activated pathway [6]. Recent studies show that calpains, caspase-3, caspase-8 and caspase-9 are all up-regulated in experimental retinal detachment, which suggests calpains are involved in caspase-dependent photoreceptor death [7]. Pharmacological inhibition of phosphodiesterase 6 (PDE6) induces retinal degeneration in rod and cone-enriched retinal explants with activation of caspase-3, calpain and poly (ADP-ribose) accumulation, which suggests a potential connection between calpain activation and apoptosis [8]. However, besides its role in apoptosis, a new feature of calpains has been found recently. Cellular necrosis, which is mediated by recombinant clostridium perfringens b-toxin (rCPB) occurs upon the activation of host cell calpains [9]. Another study reported that calpains may be involved in necroptosis as well [10]. Calcium-dependent calpain is activated by increasing calcium concentration in cytoplasm in N-methyl-N’-nitro-N’-nitrosoguanidine (MNNG)-treated cells. The activated calpains cleaves BID (BH3 interacting domain death agonist) to trucked BID sequent; tBID redistributes from the cytosol to mitochondria where it regulates BAX (Bcl-2-associated X protein) activation. Once activated, BAX provoked mitochondrial tAIF release and resulted in necroptosis [10,11].

High intra-ocular pressure (HIOP) is identified as one of the characteristics of glaucoma and it is the main factor that causes visual functional damage [12]. Related studies have been confirmed that elevation, volatility and continuous rise of intraocular pressure (IOP) could cause the death of retinal ganglion cells (RGCs), retinal pigment epithelium cells, *etc*. and eventually lead to vision loss [13,14]. Rapid elevation of IOP is a critical susceptible factor that causes acute glaucoma. The study indicated that RGCs necroptosis could also exist at the early stage of aHIOP (acute high intra-ocular pressure) [15]. Our further investigation suggested that up-regulation of receptor-interacting protein 3 (RIP3) might be involved in cellular mechanism of RGCs necroptosis [16]. As reported previously, RIPs is not the only cellular pathway which modulates early neuronal necroptosis, other cellular pathways may also participate in this process [17-19]. As mentioned earlier, the potential role of calpain involved in necroptosis in RGC after aHIOP is still unknown and further studies need to be done to evaluate it. Moreover, the various types of complex pathophysiological mechanisms in aHIOP-induced retinal injury, including retinal hypoxic-ischemia, accumulation of excitatory amino acid and inflammatory molecules, *etc*. needs to be considered [20,21]. But little is currently known about whether there is necroptosis in RGC under elevated hydrostatic condition, which is an initial factor in aHIOP-induced retinal injury *in vivo*, hence, we investigated the existence of necroptosis in elevated hydrostatic condition states by PI-staining and flow cytometry, and then evaluated the effect of calpain in necroptosis of RGC under elevated hydrostatic condition. We expect that the results will lead to a better understanding of the cellular mechanism of early RGC necroptosis and looking for rational interventional targeted therapy in the future.

## Methods

### Cell culture

The mouse retinal ganglion cell line (RGC-5) was contributed by Department of Ophthalmology, Second Hospital of Ji Lin University in China [22]. RGC-5 cells grew in Dulbecco’s Modified Eagle Medium (DMEM) (HyClone Laboratories, Inc. UT) and supplemented with 10% fetal bovine serum (FBS, HyClone Laboratories, Inc. UT), 100 U/ml of penicillin and 100 μg/ml of streptomycin (HyClone Laboratories, Inc. UT). The RGC-5 cells used in the experiment were within 2-3 passages post-thawed to minimize the variability in the assays based on our observations. The density of RGC-5 cells was around 80% in 6 ml culture media in 50 ml flask before EHP (elevated hydrostatic pressure).

### PCR analysis

The cells cultured in the flasks were harvested and RNA was isolated using TRIzol (Invitrogen, Carlsbad, CA). cDNA was synthesized using Thermoscript (Invitrogen, Carlsbad, CA) from 1 μg of total RNA. Each primer pair (β-actin: forward primer

5′-CAACTTGATGTATGAAGGCTTTGGT-3′

reverse primer 5′-ACTTTTATTGGTCTCAAGTCAGTGTACAG-3′.

NGF: forward primer 5′-AGAACCGTACACAGATAGCAA-3′

reverse primer 5′-TTAATGTTCACCTCGCCCAG-3′.

BDNF: forward primer 5′-AAAACCATAAGGACGCGGACTT-3′

reverse primer 5′-AAAGAGCAGAGGAGGCTCCAA-3′.

GFAP: forward primer 5′-ACGCAATTCCATTTTACCTG-3′

reverse primer 5′-AGGCCATAACTCATGCAAC-3′) was designed to cross at least one intron and was specific to the gene of interest. Negative control was used diluted water for instead. Oligonucleotide primers that recognized the housekeeping gene β-actin were used as normalized control. PCR program consisted of 94°C for 3 min, 36 cycles of 94°C for 45 s, 55°C for 50 s, and 72°C for 2 min, with a final extension at 72°C for 10 min.

### Immunofluorescence staining

The coverslips with fixed cells were washed in 0.01 M PBS for 3 min, incubated in 5% BSA and then reacted with anti-rabbit calpain antibody (Abcam, ab39170, USA, 1:500) or anti-rabbit Brn3a (Boster, BA1773, China, 1:200) and anti-mouse THY1.1 (Abcam, ab8872, USA, 1:500) overnight. Then coverslips were reacted with Cy2 or Cy3-conjugated donkey anti-rabbit or anti-mouse secondary antibodies at 1:200 (Invitrogen, USA). Counterstaining with DAPI, then covered with an anti-fading mounting medium before microscopic examination. Control RGC-5 incubated simultaneously in a conventional incubator at 37°C.

### Cell injury and ALLN or Nec-1 usage

A pressurized incubator was designed to expose the cells to an elevated hydrostatic pressure as described in Ju’s study [23]. After 2 hr exposure in this pressure system, the pressure present in three different values: 100 mmHg, 60 mmHg and 30 mmHg, cells were then moved to conventional culture incubator to recover at each recovery time point (6 hr, 12 hr and 24 hr). ALLN (Merck, Germany) and Nec-1 (Sigma-Aldrich, USA) were dissolved in Dimethyl sulfoxide (DMSO) for storage in 10 mmol/L and 1 mg/mL, which were pretreated in 10 μmol/L for 24 hr before cell injury.

### PI staining

At each recovery time point (6 hr, 12 hr and 24 hr), the coverslips were washed in 0.01 M PBS for 3 min, and incubated in 10 μg/ml PI-dye solution at 37°C. After that, cells were fixed in 4% PF (Paraformaldehyde) and washed in PBS counterstaining with DAPI, and then covered with anti-fading mounting medium before microscopic examination. Control RGC-5 was incubated simultaneously in a conventional incubator at 37°C. Quantitative analyses were conducted using approximately 20 merged images (magnification = 40×) to estimate the frequency of cell necrosis.

### Western blot

At each survival time point, cells were digested by sonication on ice in a digestion buffer [150 mM NaCl, 25 mM Tris–HCl (pH 7.4), 2 mM EDTA, 1.0% Triton X-100, 1.0% sodium deoxycholate, 0.1% SDS] containing a cocktail of protease inhibitors (Sigma, USA). Cell lysates were centrifuged at 10,000 × *g* for 20 min at 4°C. The supernatants were collected, and protein concentration was determined by Bicinnchoninic acid (BCA) assay (Pierce, USA). A total of 100 μg of protein in 62.5 mM Tris loading buffer (pH 6.8, containing 25% glycerol, 2% SDS, 0.01% bromophenol blue and 5% β-mercaptoethanol, Bio-Rad, USA) was boiled for 10 min, and loaded into each lane of 4-20% linear gradient Tris–HCl ready gel (Bio-Rad, USA). The polypeptides were electrotransferred to Trans-Blot pure nitrocellulose membrane (Bio-Rad, USA). Non-specific binding was blocked with PBS containing 5% nonfat milk (Bio-Rad, USA) and 3% bovine serum albumin (Sigma, USA). Membranes were incubated with tAIF (Santa Cruz biotechnology Inc, SC-113116, USA, 1: 200), β-tubulin (Abcam, ab6046, USA, 1:1000), calpain (Abcam, ab39170, USA, 1:500) or actin (Abcam, ab3280, USA, 1:500) antibodies overnight, and then in HRP-conjugated secondary antibodies (1:20000, Bio-Rad, USA) for 1 hr. Immunoblotting products were visualized with an ECL Plus™ Western Blotting Detection kit according to manufacturer’s instruction (GE Healthcare Life Sci., USA), and images were captured in a Molecular Dynamics Phosphor imager (Nucleo Tech Inc., USA). Western blot bands were measured with Image J (National Institutes of Health, USA) to analyze the integrated density value (IDV). The average IDV values of tAIF or calpain with β-tubulin and actin were compared, and the average relative value was obtained.

### Flow cytometry

The cells attached to the flasks were trypsinized followed by a gentle wash. Resuspending the cells in 200 μl of 1× binding buffer, and then added 5 μl of 20 μg/ml Annexin V and 10 μl of 50 mg/ml PI, incubated at RT for 15 min in the dark. After the cells were washed and analyzed by FACS Calibur (Becton, Dickinson Company, USA). The percentages of cells in each quadrant were analyzed using ModFit software (Verity Software House Topsham, USA). Statistical results of flow cytometry were conducted by calculating the PI+ cells numbers. All the results were repeated for three times.

### Calpain activity assay

Calpain activity was determined by cleavage of the substrate Ac-LLY-AFC (Abcam, USA). The attached cell were digested, and then centrifuged for 1 min in a microcentrifuge (10,000 × *g*) then the supernatant was transferred to a fresh tube, a part of the supernatant were used to measure the protein concentration. The fluorescence was measured after 60 min incubation at 37°C along with the substrate in the reaction buffer. Fluorescence was recorded in a Fluoroskan Ascent Fluorimeter (Labsystems, Eragny-Parc, France), and the final results were expressed as Relative Fluorescent Unit (RFU) per milligram protein of each sample.

### Data analysis

Figure panels were assembled by using Photoshop CC. The data were analyzed by using SPSS 19.0 (SPSS, USA). One-way analysis of variance (one-way ANOVA) was performed to test differences in average value between groups. All results were presented as mean ± SD. A value of *p* < 0.05 was considered statistically significant.

## Results

### RGC-5 has the same features with retinal ganglion cells

**Figure 1:**
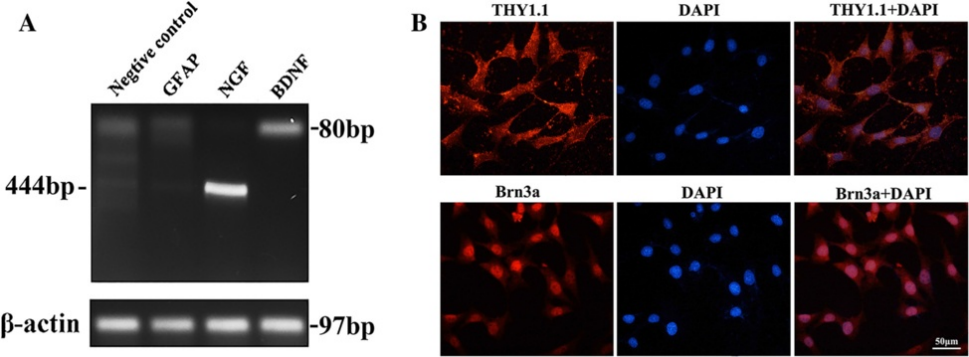
**Identification of RGC-5 cell line.** Panel **A**: RT-PCR show retinal ganglion cell marker gene expressed in our cultured cell, Panel **B**: Two other types of protein marker also detected with immunostaining, scale bar = 50 μm.

First we verified that the RGC-5 cells in our culture conditions expressed BDNF and NGF, but not GFAP (Figure 1A). As shown in Figure 1B, the cells expressed RGC marker Brn3a and Thy1.1. The transcription factor Brn3a was expressed and appears to localize at the nuclei of RGC-5 cells. On the other hand, the expression of Thy 1.1 was detected in the cytoplasm of RGC-5 cells. Negative control immunostaining (second antibody only) showed no positive signal (data not shown), confirming the specificity of the antibodies. Therefore, expression of RGC marker genes and protein indicated that the cells cultured were RGCs.

### Necroptosis happened at early stage of EHP

**Figure 2:**
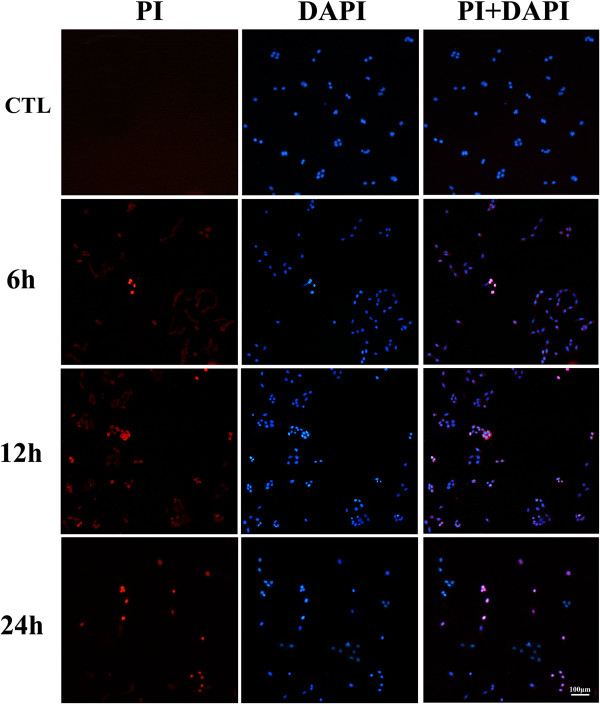
**Quantitative analysis of necrosis cells in low pressure.** PI/DAPI staining in 60 mmHg, scale bar = 100 μm.

**Figure 3:**
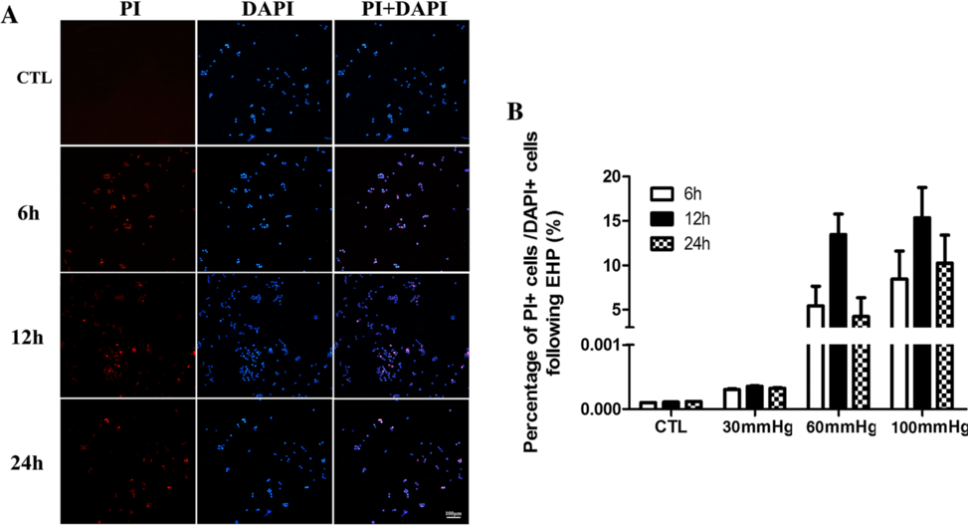
**Quantitative analysis of necrosis cells in high pressure.** Panel **A**: PI/DAPI staining in 100 mmHg, scale bar = 100 μm; Panel **B**: The statistical analysis of RGC-5 necrosis.

Propidium iodide (PI) is one of the dyes that emit red fluorescent under the excitation of 535 nm with the combination of DNA double strain through necrosis cell’s membrane, but it does not penetrate into live cells. Thus, PI dye can be used to distinguish the necrosis cell from normal ones [24-27]. DAPI is one of the dyes that emit blue fluorescent under the excitation of 340 nm with the combination of DNA double strain through 4% PF fixation. It can identify PI-staining to be a realistic cell but not false-positive dying [28]. The double labeling of PI and DAPI showed that there was no significant PI staining in 30 mmHg insults (data not shown). With the counterstaining of DAPI, a few PI-positive cells were observed in the condition of 60 mmHg (Figure 2). In our observation time point, the number of PI-positive cells gradually increased to 13.45% in 12 hr and decreased to 4.32% afterward in 24 hr (Figure 3B). In the condition of high pressure in 100 mmHg, necrosis also occurs at 6 hr (Figure 3A). The number of PI-positive cells reached 15.35% at 12 hr, and gradually decreased at 24 hr (Figure 3B). These results indicated that within our observation time point, there was a small number of necrosis in RGC-5 cell line under 60 mmHg and the degree of cell necrosis increased following the pressure elevation (Figure 3B). During the same time point, we observed that as the pressure level elevated, the number of necrosis cells increased too. This result indicates that RGC-5 necrosis is affected in a pressure-dependent manner. Correspondingly, it is suggested that under 100 mmHg there is more necrosis cells in RGC-5. In order to be consistent with the conditions *in vivo*[29], we believed that 100 mmHg insult condition was suitable for the next step of our experiment for necroptosis detection.

**Figure 4:**
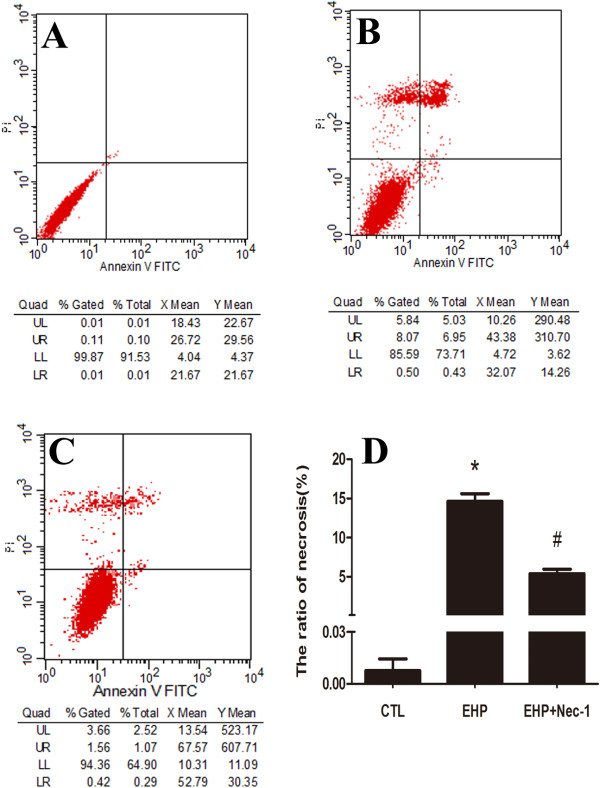
**Quantitation of necrotic cells under hydrostatic pressure and Nec-1 usage.** Panel **A**: Normal control cells; Panel **B**: RGC-5 cell lines necrosis under elevate hydrostatic pressure; Panel **C**: RGC-5 cell lines were treated with necrostatin-1 (10 μmol/L) to block necroptosis for 24 hr in cell density of 0.6 × 10^5^/ml before elevate hydrostatic pressure and analysis of necroptotic cells. Cells were stained with Annexin FITC and PI, and analyzed by FACS using FL1 (Annexin) and FL3 (PI) channels. Panel **D**: The statistical analysis of RGC-5 necrosis, * *vs* # *p* < 0.05.

It’s difficult to precisely identify between necroptosis, apoptosis or necrosis in various methods until recently [30]. Nec-1 is the specific inhibitor of necroptosis, as reported in other studies the cell number of necrosis decreased when using it [31,32]. In order to analyze necroptosis, we detected the PI-positive cells to analyze cellular necroptosis by flow cytometry with PI/Annexin V double staining using Nec-1. In our model of cell injury in 24 hr in 100 mmHg, we found that the ratio of necrosis cells (PI+) was about 13% (Figure 4B), but the percentage decreased to nearly 5% (Figure 4C) within the use of Nec-1 which have significant difference in statistical analysis (Figure 4D). These results indicate that with the exposure of Nec-1, necrosis is inhibited, thus necroptosis occurred after EHP.

### Calpain is up-regulated following elevated hydrostatic pressure

**Figure 5:**
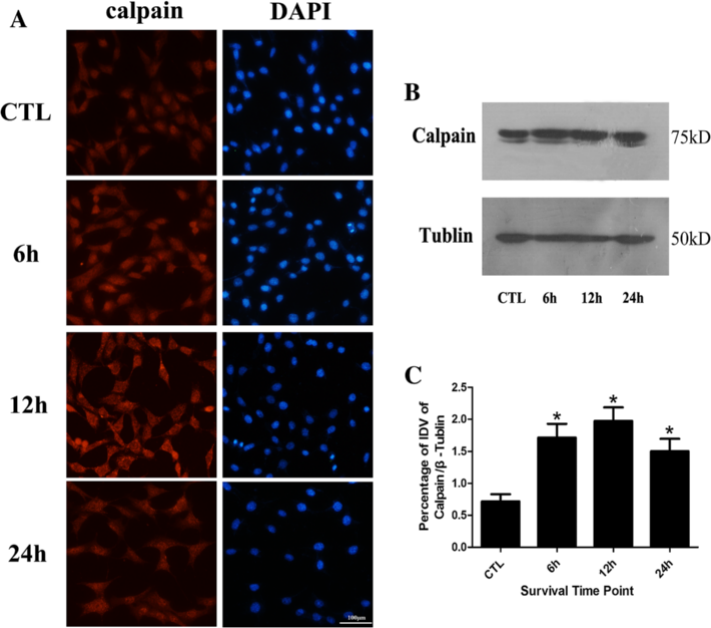
**Calpain protein expression level detection.** Panel **A**: Immunofluorescence staining of calpain; Panel **B**: Western blot of calpain after cell injury; Panel **C**: The statistical analysis of calpain western blot bands; *: 6 hr, 12 hr and 24 hr group *vs* CTL group: *p* < 0.05, scale bar = 100 μm in panel **A**.

Immunofluorescence staining results showed that calpain is mainly present in cytoplasm in normal control group in RGC-5 (Figure 5A); meanwhile, no difference in distribution was observed between injury group and normal controls. Generally, in contrast with the normal controls, significantly more distinct and heavier calpain immunoreactive was found with strong cellular labeling of calpain in 6 hr and 12 hr groups in high pressure models, but weaker labelling in 24 hr group. The western blot results showed that calpain exhibited mainly as a single 75 kDa band in all groups (Figure 5B). The bands in high pressure groups were apparently thicker and larger than those of normal control groups. The bands in 24 hr group were thinner and smaller than those in injury groups and tended to be normal. Statistical analysis of IDV indicated that high pressure up-regulated the expression of calpain in the early stage (Figure 5C), significantly more distinct calpain bands were shown in 12 hr injury group (*p* < 0.05). It demonstrated that calpain distribution have no difference between injury groups and normal controls, the protein expression levels increased at first and then decreased as time extend within 1 day, which reached the maximum detection in 12 hr group.

### AIF cleavage product decreased after calpain inhibition

**Figure 6:**
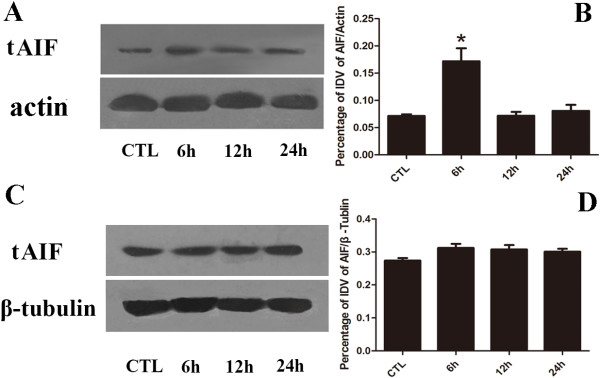
**tAIF protein expression level detection.** Panel **A**: Western blot of tAIF after cell injury; Panel **B**: The statistical analysis of tAIF western blot bands after injury; Panel **C**: Western blot of tAIF after cell injury pretreated with ALLN; Panel **D**: The statistical analysis of tAIF western blot bands after calpain inhibition. *: 6 hr group *vs* other groups: *p* < 0.05.

At first, the bands of tAIF in high pressure groups of 6 hr, 12 hr and 24 hr increased and then decreased as time extended the maximum detection in 6 hr group (Figure 6A). The statistical analysis of IDV suggested that high pressure up-regulated the cleavage of tAIF in the early stage (Figure 6B), significantly more distinct tAIF band were shown in 12 hr injury group (*p* < 0.05). After ALLN addition, no significant changes of tAIF band in all four groups were detected (Figure 6C), the statistical analysis of IDV also confirmed about these results (Figure 6D), which indicated that there is no difference in protein expression levels in tAIF production in all groups.

### Calpain activity assay

**Figure 7:**
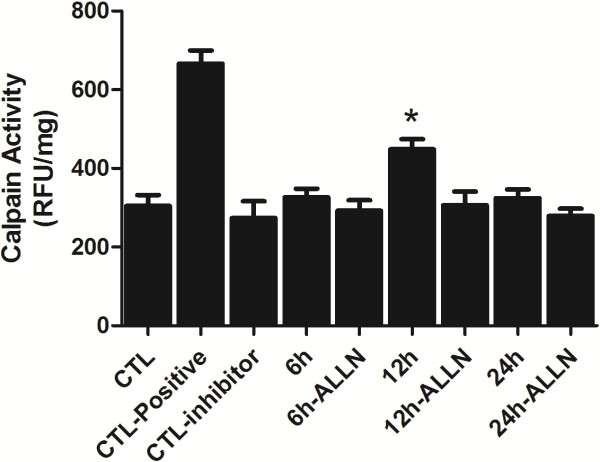
**The statistical analysis of calpain activity assay.** CTL: Normal control RGC-5; CTL-positive: Normal control RGC-5 cell lysate with active calpain addition according to the detection kit; CTL-inhibitor: Normal control RGC-5 cell lysate with Z-LLY-FMK addition according to the detection kit; 6 hr, 12 hr and 24 hr means the survival time point after cell injury; 6 h-ALLN, 12 h-ALLN and 24 h-ALLN means pretreated with ALLN for 24 hr before cell injury; *: 12 hr group *vs* 12 hr ALLN treatment group: *p* < 0.05.

With both the inhibitor and injury groups preteated with ALLN for 24 hr, the calpain activity assay appeared to be higher in the inhibitor group (Figure 7). However, at 12 hr there were significant changes between the inhibitor and injury group (*p* < 0.05). It suggested that ALLN may effectively inhibit up-regulation of calpain activity following hydrostatic pressure treatment.

### ALLN may decrease the rate of RGC-5 necrosis

**Figure 8:**
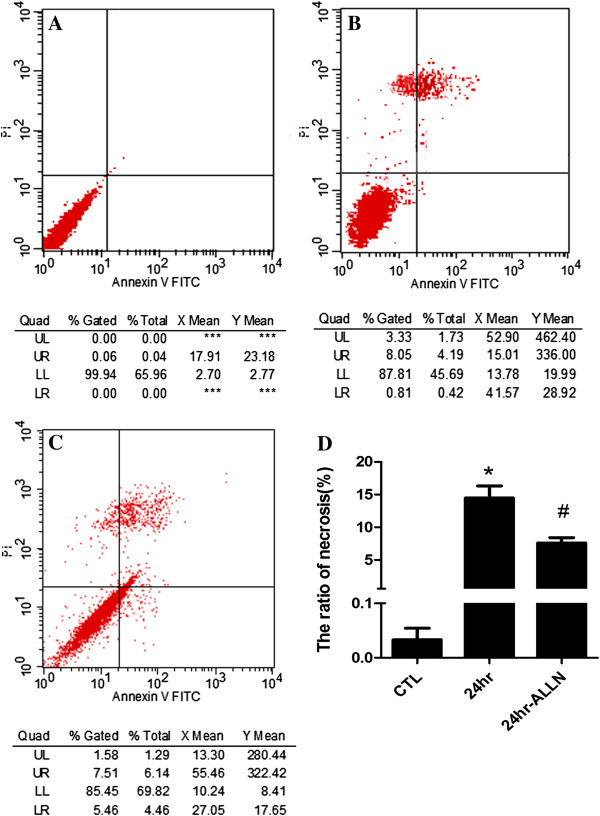
**Quantitation of necrotic cells under hydrostatic pressure and calpain inhibition.** Panel **A**: Normal control cells; Panel **B**: RGC-5 cell lines necrosis under elevate hydrostatic pressure; Panel **C**: RGC-5 cell lines were pre-treated with ALLN to inhibit calpain activity for 24 hr before elevating hydrostatic pressure; Panel **D**: The statistical analysis of RGC-5 necrosis. Cells were stained with Annexin FITC and PI, and analyzed by FACS using FL1 (Annexin) and FL3 (PI) channels. *: 24 hr group *vs* CTL or 24 hr-ALLN treated group: *p* < 0.05.

Under 100 mmHg insult, our study showed the occurrence of necroptosis in RGC-5 for 2 hr but recovery at 24 hr. Therefore, the cells were treated under this condition with the addition of ALLN. After that, we analyzed cellular necroptosis by using flow cytometry with PI/Annexin V double staining to detect whether it could decrease the rate of necrosis in RGC-5 under high pressure condition after calpain activity has been inhibited. The results showed that the ratio of necrosis cells is about 12% (Figure 8B), the percentage decreased to nearly 8% upon adding ALLN (Figure 8C) in the injury group in 24 hr. Similar with Nec-1 treatment, it demonstrated that the ratio of RGC-5 necrosis decreased when treated with ALLN under high pressure condition. Meanwhile, statistical analysis indicated that there were significant changes in PI-positive cells upon adding ALLN compared to normal control group and EHP group (Figure 8D), these results indicated that RGC-5 necroptosis in the early stage may be related to the up-regulated calpain activity.

## Discussions

HIOP is one of the main features and risk of visual impairment with ganglion cell death in glaucoma [33-35]. Some research demonstrated that acute increase or continuous arise of intra-ocular pressure (IOP) could lead to visual injury or cell death in retinal ganglion cells, pigment epithelial cells and finally resulting in vision lost. Particularly, retinal ganglion cell death is the most important behavior [36,37] in acute glaucoma. The pathological research model of acute glaucoma is divided into animal model and cell model [16,38,39]. Elevated hydrostatic pressure (EHP) in cultured cell line is commonly used in glaucoma cell model *in vitro*[40]. In this study, we used open cycling air pressure culture system to gain hydrostatic pressure, the system could set pressure values as required and may easily gain more information *via* high-throughput experiments [41].

In our study, we chose three pressure values (100 mmHg, 30 mmHg and 60 mmHg) to detect possible levels in necrosis after injury. Among these three pressure values, 100 mmHg represented acute glaucoma (high pressure), 30 mmHg represented low pressure in glaucoma [40] and 60 mmHg was a maximum pressure value in human IOP [42]. The results indicated that the number of PI positive cells increased first and then decreased followed by the time passage in 100 mmHg and 60 mmHg. This is consistent with our previous study on rat (110 mmHg for 1 hr) [16], but there was a small number of PI-positive cells in 30 mmHg (data not shown). It was inconsistent with the chronic glaucoma mouse model following 20-30 mmHg [43], one possibility may be that glaucoma is a neuronal degenerative disease; the symptom might take a longer time to exhibit. Therefore, the fact that having a few obvious necrosis cells in our acute insult for a short time seems to be reasonable. Joo’s study have found that necrosis cells existed in ganglion cell layer in 160-180 mmHg insult for 90 min following HIOP at 4 hr, but a few necrotic cell was observed after 24 hr [44], but we found some necrosis cells existed in 24 hr, it might be due to the higher pressure and also cells being kept longer in their study. It may also be related to the complexity in the micro-environment (neighboring cells and the secretion of various factors in interstitial cells). Our experiment demonstrated that cell model after EHP could accurately reflect the injury degree of RGCs. Moreover, Nec-1 has become one of the widely recognized inhibitors in necroptosis [31]. Based on our data, the necroptosis can be found after high pressure insult (100 mmHg), and the proportion was reflected by the fact that the necrosis rate decreased to about 5% after adding Nec-1. Therefore, we speculated one signaling molecules that may induce necroptosis of RGC-5 after EHP.

Previous studies have shown that beside calpain mediated apoptosis, calpain can also induce necroptosis through intracellular signal pathway. Calpain facilitates BAX activation and activated BAX favors the release of tAIF from mitochondria to the cytosol which could induce necroptosis [10,11]. Calpain may be one of the important regulatory molecules participating in necroptosis in the cells like fibroblast, nephrocyte, HeLa cell and vascular endothelial cells, *etc*. Some early necroptosis may be mediated by calpain [9-11,45]; however, the biological function of calpain-mediating early neuronal necroptosis in nervous system especially the visual nervous system is largely unveiled. Our western blot results showed that calpain protein levels appeared to decrease after an initial increase, and reached maximum at 12 hr following elevated hydrostatic pressure condition in RGC-5. In this study, the immunofluorescence staining results showed that calpain was mainly present in the cytoplasm of RGC-5. Moreover, in contrast with other groups, significantly more distinct and heavier calpain immunoreactive were shown in 12 hr group. Both of these results showed that the expression of calpain was significantly up-regulated in RGC-5 following elevated hydrostatic pressure condition accompanied with the phenotype of the increased ratio of necroptosis. This observation in regards to the expression profile of calpain consists of what we found in gene chip detection in RGC-5 following elevated hydrostatic pressure in our previous experiment (our previous unpublished data). In contrast, necroptosis was reduced to a certain extent when exposed to ALLN, a specific calpain inhibitor targeting the activity rather than the expression [46], measured by flow cytometry. Collecting all these results together, it suggested that calpain may play an important role in early RGC-5 necroptosis following elevated hydrostatic pressure condition.

In our experiment, with ALLN intervention the expression of calpain remained high while the activity was markedly inhibited in RGC-5 following elevated hydrostatic pressure condition. Moreover, tAIF (the cleaved form of AIF) didn’t significantly increase, which resulted in less effect on RGC-5. Meanwhile, the number of necroptosis cells didn’t significantly increase either. It further demonstrated that calpain in RGC-5 was mediated by the downstream molecule of tAIF to modulate necroptosis pathway. Overall, the necroptosis regulated by calpain mediated by tAIF may be a mode of RGCs death induced by elevated hydrostatic pressure. Regarding to another risk factor, cellular hypoxic exposure is not only the widely used nervous system injury model [47], but also one of the important pathophysiological mechanisms of aHIOP [48]. Therefore, it is worthwhile to further investigate whether necroptosis is mediated by calpain in hypoxic exposure. It will be helpful for comprehensive understanding about RGC-5 necroptosis in aHIOP. Taken all together, the result provided novel evidence for the molecular mechanism (non-RIP3 pathway) research of the early RGCs in aHIOP and new interventional target for reducing early RGCs necroptosis in aHIOP patients.

## Conclusion

Our study found that calpain may induce necroptosis *via* tAIF-modulation in RGC-5 following elevated hydrostatic pressure.

## Competing interests

The authors declare that they have no competing interests.

## Authors’ contributions

LS, KX and JFH designed the experiment, LS and KX performed the experiment, WD and SC analyzed the data, LS, RFM and KX drafted the manuscript, LXX participated in paper modification and revised the manuscript for English writing, all authors participated in critical revision of the manuscript and approved the final manuscript.
